# “Revivendo Memórias” a passion Reminiscence Therapy intervention for People with Cognitive impairment that live in Nursing Homes in Brazil: characteristics of participants of a pilot study

**DOI:** 10.1002/alz70858_107739

**Published:** 2025-12-25

**Authors:** Michele Gomes Ferreira, Carlos Chechetti, Maria Luiza dos Santos Barbosa, Carla Beatriz Gondim, Carla Aparecida Arena Ventura, Sonia Maria Dozzi Brucki, Leonel Tadao Takada

**Affiliations:** ^1^ Federal University of Minas Gerais, Belo Horizonte, Brazil; ^2^ Associação Revivendo Memórias, São Paulo, São Paulo, Brazil; ^3^ Cognitive and Behavioural Neurology Unit, Hospital das Clínicas, University of São Paulo, São Paulo, São Paulo, Brazil; ^4^ Universidade de São Paulo, Ribeirão Preto, São Paulo, Brazil

## Abstract

**Background:**

*Revivendo Memórias* (RM) is a social program that leverages personal passions as a cognitive and socialization activity for older adults, people with mild cognitive impairment, and people living with dementia. The methodological framework of RM is based on reminiscence therapy, using materials that connect people with their passions, which include music, movies, sports, etc. Since 2019, RM has been implemented in Brazilian museums, and this study explores its feasibility in nursing homes.

**Method:**

The study was conducted in three nursing homes across Brazilian cities: São Paulo, Belo Horizonte, and Ribeirão Preto. In each location, 10 participants with cognitive decline were selected (*N* = 30). The intervention consisted of four one‐hour group reminiscence therapy sessions. Sessions incorporated personalized passion‐related materials, reference cards featuring music, television programs, and soccer. A sociodemographic questionnaire was completed with the assistance from nursing home professionals. Additionally, participants were assessed using standardized instruments, including the Mini‐Mental State Examination (MMSE).

**Results:**

Participants were predominantly male (56.7%), with a mean age of 75.33 years (SD = 7.69) and an average of 6.57 years of formal education (SD = 3.63). The mean length of stay in the nursing home was four years. Regarding marital status,50% of the participants were single, 30% divorced, 13% widowed and 6.7% married. MMSE scores ranged from 3 to 25 (*M* = 15.63, SD = 6.73). Although participants had cognitive decline, only 53,3% of them had a formal diagnosis. According to the Functional Assessment Staging (FAST) scale, completed by the staff, 10% of the participants were classified as normal adults, 16,7% normal aged person, 33% mild Alzheimer´s Disease (AD), 33% moderate AD and 6,7% severe AD.

**Conclusion:**

People that live in Nursing Homes may benefit from RM intervention, however, it is important to understand the population that live in residential care facilities. The current study showed that participants have low level of education and access to diagnosis was difficult. The material used in Nursing Homes may need to be adapted according to the cognitive level of the participants.

